# Mode of birth and women’s psychological and physical wellbeing in the postnatal period

**DOI:** 10.1186/1471-2393-12-138

**Published:** 2012-11-28

**Authors:** Ingrid J Rowlands, Maggie Redshaw

**Affiliations:** 1 Gynaecological Cancers Group, Queensland Institute of Medical Research, 300 Herston Road, Herston, Brisbane, 4006, Australia; 2 Policy Research Unit for Maternal Health and Care, National Perinatal Epidemiology Unit, University of Oxford, Old Road, Oxford, OX3 7LF, UK

**Keywords:** Childbirth, Mode of birth, Postpartum, Posttraumatic stress, Maternal health

## Abstract

**Background:**

Physical and psychological problems after childbirth are common, and may have a significant negative and long-term impact on women’s wellbeing and daily functioning. The method of birth may be a particularly important factor influencing women’s health and wellbeing following birth, however, population-wide evidence is limited. This study uses data from 5,332 women who responded to a national survey of women’s experiences of maternity care in England. We examined women’s postnatal wellbeing in the first three months after birth, and whether these varied by mode of birth.

**Methods:**

This is a secondary analysis of survey data using a random sample of women selected from birth registration. We used multinomial logistic regression models to examine the association between women’s self-reported psychological symptoms, health problems and mode of birth.

**Results:**

Women who had forceps-assisted vaginal births and unplanned caesarean section births reported the poorest health and wellbeing, while those of women who had unassisted vaginal births and planned caesarean section births were less affected by the birth process. Most women’s physical and emotional health appeared to improve with time, however, those who had a forceps-assisted vaginal birth were more likely to report ongoing posttraumatic-type symptoms several months after the birth.

**Conclusions:**

Mode of birth was associated with differences in outcomes at three months. By comparison to women who had unassisted vaginal births, the risk of reduced postnatal health and wellbeing was higher amongst the women who had forceps-assisted vaginal births but not amongst women who had ventouse-assisted vaginal births. This would suggest that it is important to differentiate the different types of instrumental birth in outcome studies. Of concern was the higher rate of posttraumatic-type symptoms among women who had forceps-assisted vaginal births relative to the other modes of birth. Women who have forceps-assisted births should be monitored carefully by health professionals in the postnatal period, and in the months after childbirth, when they could be offered the opportunity to discuss their labour and birth.

## Background

Physical and psychological problems after childbirth are common, and may have a significant negative and possibly long-term impact on women’s wellbeing and daily functioning [1]. One factor that has been more consistently identified as influencing the duration and severity of women’s physical and psychological symptoms following childbirth is the mode of birth. However, population-wide evidence to demonstrate this association is largely lacking; the quality of the research varies considerably and few studies have examined both women’s psychological and physical outcomes and explored the possibility that these symptoms may influence one another. Although findings from earlier studies related to postnatal outcomes and mode of birth were based on relatively large samples [2,3], subsequent studies have relied on small samples, and there are few studies that have been able to separate the psychological and physical outcomes for women who have forceps-assisted births from ventouse-assisted births. A recent large, population-based study from Norway, focusing on antenatal and postnatal emotional health, did not differentiate between forceps and ventouse-assisted vaginal births and found no association with mode of birth [4].

Perineal problems, backache, pain during intercourse, and extreme tiredness are common problems for women during the first three months after birth [5,6]. Although caesarean section births have been identified as contributing to reductions in women’s postnatal physical wellbeing, more recently there has been interest in exploring the impact of assisted vaginal births. In the first few months after birth, assisted vaginal births have been associated with a greater likelihood of perineal pain [7,8] urinary incontinence, haemorrhoids, and sexual problems [7,9-11] than unassisted vaginal births, even when taking into account aspects related to the birth such as the length of labour and degree of perineal trauma [7,11]. Women who have assisted vaginal births, particularly forceps-assisted vaginal births may also be at risk for ongoing and continuing physical problems [12]. A recent longitudinal study from Birmingham found that faecal incontinence at 12 years postpartum was increased among women who had a birth history that included a forceps-assisted vaginal birth, but not with a birth history that included a ventouse-assisted vaginal birth [13].

Obstetric intervention, particularly emergency caesarean section, has also been associated with depressive symptoms after birth, however, results are inconsistent [14] and recent evidence from large cohort studies has found no association [4,15,16]. More consistently, caesarean section births have been associated with feelings of dissatisfaction related to the birth and with poor body image and lower self-esteem [14], possibly increasing the risk of depression in the longer term. Recently, there has been an emergence of studies focusing on anxiety disorders, particularly posttraumatic stress disorder (PTSD) after childbirth, which occurs in a small proportion of women (1-6%) [17]. Studies show that the risk of postnatal PTSD symptoms is higher for women who have had unplanned caesarean section births followed by women who have had assisted vaginal births [18-20]. However, other researchers have found that women who have had an assisted vaginal birth are more likely to perceive the birth as “extremely distressing” six weeks after birth than women who have had a caesarean section or unassisted vaginal birth [21]. This study, however, did not differentiate between forceps and ventouse-assisted births and this may be important for understanding the influence of mode of birth on postnatal PTSD-type symptoms.

The experience of childbirth can be complex due to a wide range of individual, medical and social factors that can interact to influence women’s experiences and outcomes. This study examines the physical and psychological outcomes of women in the first three months after birth, and whether these varied by mode of birth. We specifically examined whether women who had assisted vaginal births and operative births experienced poorer psychological and physical health than women who had unassisted vaginal births. Because women’s physical symptoms after birth may affect the severity and duration of their self-reported psychological outcomes and vice versa [22], we adjusted for concurrent psychological and physical symptoms in our analyses when examining women’s postnatal outcomes. The results of this study will provide important information for health professionals about those women who may be in greater need of support during and after childbirth.

## Methods

### Participants

The study used data collected as part of the National Maternity Survey 2010, which aimed to document women’s experiences of maternity care in England, to describe current practices, the care received and changes in these since the last survey conducted in 2006 [23,24]. Based on birth registration, surveys were sent by the Office for National Statistics to a random sample of 10,000 women aged 16 years and over, who had recently given birth in England over a two week period. Questionnaires were mailed out in January 2010 at three months postpartum, and women were asked about their experiences during pregnancy, birth and during the postnatal period. Two methods of responding to the survey were offered: a paper questionnaire returned by post or an online survey. Tailored reminders were sent out: a letter after two weeks, a further questionnaire after four weeks and a further reminder letter four weeks after that.

### Measures

The National Maternity Survey was designed to document the care and support provided to women during pregnancy, birth and after birth. Women were also asked about their health and wellbeing over this time, about partner engagement and support for infant care. Demographic information including age, parity, ethnicity, age left full-time education, marital status, employment status, Index of Multiple Deprivation (IMD) (a small area based measure) and country of birth were also collected. Mode of birth was self-reported and categorised as five groups: unassisted vaginal birth, ventouse-assisted vaginal birth, forceps- assisted vaginal birth, unplanned caesarean-section and planned caesarean-section.

Women’s psychological and physical wellbeing after birth were assessed using a checklist of common postnatal psychological and physical symptoms based on that used in earlier national maternity surveys [25]. Women were asked, “Did you experience any of the following…” and chose from a list of physical problems and psychological symptoms: “the blues”, painful stitches, breastfeeding problems, depression, wound infection, stress incontinence, fatigue or severe tiredness, backache, anxiety, pain during intercourse, sleep problems not related to the baby, flashbacks to the labour and birth and difficulties in concentrating. At 10 days, 1 month and 3 months, women were asked to tick the box for each symptom if the symptom was present at that particular time point after the birth.

The study undertaken was carried out as a secondary analysis of survey data. The original survey on which the evaluation of maternity services in England was based was passed by the Trent Multi-Centre Research Ethics Committee.

Exploratory factor analysis, namely principal components analysis, was conducted to determine the factor structure underlying the postnatal symptoms. We specified an oblique promax rotation to account for the correlations among the subscales. Initial eigen values for the first four factors were all above 1, which was reflected in the scree plot where values “levelled off” after first four factors, suggesting a four factor model. The first four factors explained 47% of the total variance, with each factor explaining 22%, 9%, 8% and 8% of the variance, respectively. The factor loadings showed that the psychological symptoms loaded highly on the first factor and the three PTSD items clustered with these items. The physical symptoms: “stress incontinence”, “backache” and “difficulties/pain during intercourse” loaded highly together on the second factor, while “breastfeeding difficulties” loaded on the third factor and “painful stitches” and “wound infection” loaded together on the fourth factor. Severe tiredness/fatigue did not appear to load highly with any of the other items, however, as this was an important and prevalent postnatal symptom, this variable was included in our analysis.

Based on the results of the factor analysis, psychological theory and literature [26-28], the items were grouped as follows: psychological symptoms (e.g. “the blues”, depression, anxiety); posttraumatic stress-type symptoms (e.g. flash-backs, difficulties concentrating, sleep problems not related to the baby); bodily changes (e.g. stress incontinence, backache; difficulties/pain during intercourse); birth-related symptoms (e.g. painful stitches, wound infection); breastfeeding problems and severe fatigue. All items in each grouping were combined to form a single variable representing those items, except for the psychological symptoms, breastfeeding problems and severe fatigue, which were examined separately.

### Statistical analysis

Univariate analyses (analysis of variance, chi-square tests) were used to test for associations between demographic and obstetric characteristics, psychological and physical symptoms and mode of birth at 10 days, 1 month and 3 months. We used multinomial logistic regression models to examine whether women’s psychological and physical outcomes at the three time points varied according to the mode of birth. The models were adjusted for key sociodemographic variables including mother’s age (in years), parity (primiparous, multiparous), age on leaving full-time education (<16, 17–18, ≥19), Index of Multiple Deprivation (IMD; quintiles), ethnicity (white, other) and all other symptoms. Although univariate and multivariate analyses were conducted to examine the differences between mode of birth and psychological and physical symptoms at all three time points, we have presented the results for 1 month and 3 months only, as these time points are most likely to be associated with longer-term problems*.* All statistical analyses were performed using SPSS version 19.

## Results

### Participant characteristics

Of the 5,332 women who responded to the survey, more than half (58%) were between the ages of 25 and 34 years. The median age of babies at the time of women responding was 3.1 months (mean 3.3 months, s.d. 0.65 and range 2.2-6.6 months). Half the women had given birth before, 12% were single parents at the time of the survey, 22% had left school by the age of 16 years, 14% were from Black and Minority Ethnic Groups and 21% had been born outside the UK. Data on women who did not respond to the survey were provided by the Office of National Statistics and comparisons showed that non-respondents were more likely to be younger, living in a more disadvantaged area and from a Black and Minority Ethnic background [23].

Table 1 shows the characteristics of the women included in the analysis according to mode of birth. Most women (*n* = 3275, 61%) had unassisted vaginal births. Ventouse-assisted vaginal births and forceps-assisted vaginal births were reported less frequently by 6% (*n* = 302) and 7% (*n* = 359) of women, respectively. A total of 12% (*n* = 630) of women had a planned caesarean and for 13% (*n* = 675) their caesarean was unplanned. Women who had unassisted vaginal births were more likely to be younger, not married, to have previously given birth and less likely to have been in fulltime education after the age of 19 years. In contrast, women who reported having a ventouse-assisted vaginal birth were more likely to be married, to have recently given birth to their first baby, to have received further education after the age of 19 years and to be from a white ethnic background. Women who had a forceps-assisted vaginal birth were similar to women having a ventouse-assisted vaginal birth on marital status, parity and ethnicity but they were less likely to have received any further education after the age of 19 years. Women reporting an unplanned caesarean were also similar to women who had had assisted vaginal births on marital status and parity but they were more likely to be from a black minority ethnic background and not born in the UK. Women who had planned caesareans were more likely to be older, married and to have previously given birth than women delivering by other methods.

**Table 1:** Characteristics of women in the national maternity survey by mode of delivery

	**Non-operative births**			**Operative births**		
	**Unassisted Vaginal**^**a**^	**Ventouse-assisted**^**a**^	**Forceps-assisted**^**a**^	**Unplanned Caesarean**^**a**^	**Planned Caesarean**^**a**^	***p***
	**n = 3275**	**n = 302**	**n = 359**	**n = 675**	**n = 630**	
	**n (%)**	**n (%)**	**n (%)**	**n (%)**	**n (%)**	
**DEMOGRAPHIC**						
**Mother’s age (years)**						
M (SD)	30.11 (5.75)	30.27 (5.26)	30.30 (5.49)	31.07 (5.55)	32.88 (5.31)	<0.001
**Parity**						
Primiparous	1342 (42)	246 (83)	299 (83)	492 (75)	193 (32)	<0.001
Multiparous	1880 (58)	51 (17)	51 (15)	164 (25)	420 (69)	
**Ethnicity**						
White	2780 (86)	279 (93)	322 (90)	534 (81)	521 (84)	<0.001
Other^b^	445 (14)	20 (7)	34 (10)	129 (20)	96 (16)	
**Age left full-time education (years)**						
≤ 16	751 (23)	51 (17)	63 (18)	115 (17)	151 (25)	<0.001
17-18	892 (28)	60 (20)	98 (28)	173 (26)	155 (25)	
≥ 19	1583 (49)	189 (63)	195 (55)	377 (57)	309 (50)	
**IMD (quintiles)**						
1^st^ (least deprived)	640 (20)	68 (23)	75 (21)	122 (18)	141 (18)	0.002
2^nd^	652 (20)	63 (21)	71 (20)	110 (16)	137 (22)	
3^rd^	681 (21)	59 (20)	74 (21)	152 (23)	151 (24)	
4^th^	629 (19)	68 (23)	62 (17)	152 (23)	85 (14)	
5^th^ (most deprived)	672 (21)	44 (15)	77 (21)	139 (21)	116 (18)	
**Marital status**						
Married	195 (59)	195 (65)	225 (63)	423 (63)	439 (70)	<0.001
Cohabiting	998 (31)	84 (28)	107 (30)	184 (27)	153 (24)	
Other	330 (10)	23 (8)	27 (8)	68 (10)	38 (6)	
**Country of birth**						
UK	2498 (80)	238 (81)	286 (83)	482 (75)	456 (77)	0.005
Other	633 (20)	55 (19)	59 (17)	165 (26)	137 (23)	
**OBSTETRIC**						
**Monitoring of baby in labour**						
No^c^	269 (8)	2 (0.7)	4 (1)	88 (13)	33 (27)	<0.001
Stethoscope/sonicaid	845 (26)	23 (8)	23 (6)	23 (4)	3 (2)	
Intermittent	674 (21)	51 (17)	64 (18)	95 (14)	25 (20)	
Constant	1344 (41)	221 (73)	255 (71)	444 (67)	56 (45)	
Other	11 (0.3)	0 (0)	0 (0)	2 (0.3)	1 (0.8)	
Don’t know	128 (4)	5 (2)	13 (4)	11 (1.7)	6 (5)	
**Duration of labour (hours)**						
≤ 7	1825 (59)	64 (22)	60 (17)	------	------	<0.001
8-11	448 (15)	58 (20)	58 (16)			
12-17	356 (12)	67 (23)	71 (20)			
≥18	468 (15)	109 (37)	165 (47)			

* *p* <0.05 ** *p* < 0.01 *** *p* < 0.001.

^a^Numbers may not sum to total because some data missing.

^b^Includes Mixed, Asian or Asian British, Black or Black British, Chinese and other.

^c^Excludes women who had a planned caesarean and did not labour.

In relation to obstetric variables, women who had assisted vaginal births were more likely to have had constant electronic monitoring during labour (ventouse: *n* =221, 73%; forceps: *n =*255, 71%); to have laboured for more than 18 hours (ventouse: *n* =109, 37%; forceps: *n =*165, 47%); to have had an episiotomy (ventouse: *n* =234, 79%; forceps: *n* =337, 94%) and perineal repair (ventouse: *n* =286, 96%; forceps: *n* =352, 99%) than women who had unassisted vaginal births (all *p* < 0.001). Women who had a forceps-assisted vaginal birth were also more likely to have had a third or fourth degree tear (*n* =41, 12%) than women who had a ventouse-assisted birth (*n* =13, 5%) or unassisted vaginal birth (*n* = 141, 4%) (*p* < 0.001).

### Prevalence of symptoms

**Figure 1:**
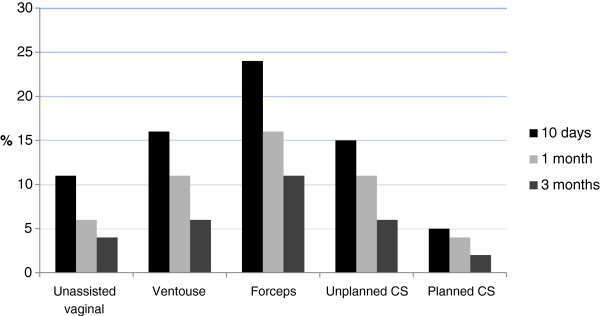
**Proportion of women reporting ‘flashbacks to the labour and birth’.** Proportion of women reporting ‘flashbacks to the labour and birth’ during the first 3 months after birth according to mode of birth.

**Figure 2:**
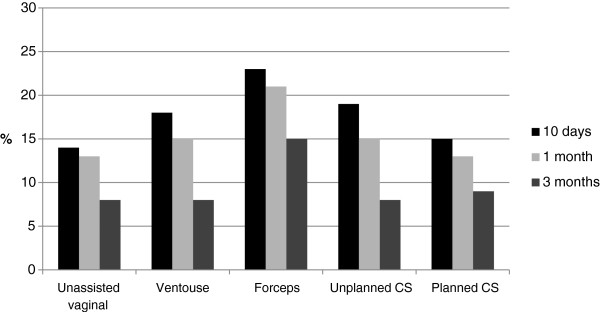
**Proportion of women reporting ‘difficulties concentrating’.** Proportion of women reporting ‘difficulties concentrating’ during the first 3 months after birth according to mode of birth.

The most common psychological symptom reported during the first 10 days was “the blues” (34%) followed by “anxiety” and “difficulties concentrating” (both 16%). While all women reported a decline in symptoms over time, PTSD-type symptoms such as “flashbacks to the labour and birth” (11 %) and “difficulties concentrating” (15%) were more common at 3 months amongst women who had a forceps-assisted vaginal birth (see Figures 1 and 2). Amongst all women the most common physical symptoms reported at 10 days after the birth were fatigue or severe tiredness (37%), breastfeeding problems (35%), painful stitches (34%) and backache (28%). As with the psychological symptoms a similar pattern of reducing prevalence over time was observed for the physical symptoms, with backache, difficulties experienced during sexual intercourse and stress incontinence occurring more commonly in women who had assisted vaginal births at 3 months (see percentages for bodily changes in Tables 2 and 3).

**Table 2:** Association between psychological symptoms, bodily changes, birth-related symptoms, breastfeeding, severe fatigue and mode of birth at 1 month after birth

	**Unassisted Vaginal**^**a**^	**Ventouse-assisted**^**a**^	**Forceps-assisted**^**a**^	**Unplanned Caesarean**^**a**^	**Planned Caesarean**^**a**^	***p***
	**n = 3275**	**n = 302**	**n = 359**	**n = 675**	**n = 630**	
	**n (%)**	**n (%)**	**n (%)**	**n (%)**	**n (%)**	
**The blues**						
No	2850 (87)	266 (88)	295 (82)	574 (85)	544 (86)	0.08
Yes	425 (13)	36 (12)	64 (18)	101 (15)	86 (14)	
**Depression**						
No	3055 (93)	289 (96)	320 (89)	627 (93)	589 (94)	0.02
Yes	220 (7)	13 (4)	39 (11)	48 (7)	41 (7)	
**Anxiety**						
No	2961 (90)	272 (90)	298 (83)	596 (88)	568 (90)	<0.001
Yes	314 (10)	30 (10)	61 (17)	79 (12)	62 (10)	
**PTSD symptoms**						
No	2646 (81)	229 (76)	243 (68)	515 (76)	529 (84)	<0.001
1	494 (15)	56 (19)	85 (24)	110 (16)	76 (12)	
≥2	135 (4)	17 (6)	31 (9)	50 (7)	25 (4)	
**Bodily changes**						
No	2156 (66)	164 (54)	202 (56)	444 (66)	453 (72)	<0.001
Yes	1119 (34)	138 (46)	157 (43)	231 (34)	177 (28)	
**Birth -related symptoms**						
No	3017 (92)	242 (80)	250 (70)	546 (81)	534 (84)	<0.001
Yes	258 (8)	60 (20)	109 (30)	129 (19)	96 (15)	
**Breastfeeding difficulties**						
No	2828 (86)	246 (82)	286 (80)	551 (82)	555 (88)	<0.001
Yes	447 (14)	56 (19)	73 (20)	124 (18)	75 (12)	
**Severe tiredness/ Fatigue**						
No	2487 (76)	222 (74)	246 (69)	488 (72)	469 (74)	0.02
Yes	788 (24)	80 (27)	113 (32)	187 (28)	161 (26)	

^a^Numbers may not sum to total because some data missing.

**Table 3:** Association between psychological symptoms, bodily changes, birth-related symptoms, breastfeeding, severe fatigue and mode of birth at 3 months after birth

	**Unassisted Vaginal**^**a**^	**Ventouse-assisted**^**a**^	**Forceps-assisted**^**a**^	**Unplanned Caesarean**^**a**^	**Planned Caesarean**^**a**^	***p***
	**n = 3275**	**n = 302**	**n = 359**	**n = 675**	**n = 630**	
	**n (%)**	**n (%)**	**n (%)**	**n (%)**	**n (%)**	
**Depression**^**b**^						
No	3050 (93)	285 (94)	328 (91)	632 (94)	591 (94)	0.53
Yes	225 (7)	17 (6)	31 (9)	43 (6)	39 (6)	
**Anxiety**						
No	3123 (95)	287 (95)	335 (93)	636 (94)	597 (95)	0.41
Yes	152 (5)	15 (5)	24 (7)	39 (6)	33 (5)	
**PTSD symptoms**						
No	2842 (87)	254 (84)	283 (79)	567 (84)	557 (88)	<0.001
1	340 (10)	43 (14)	51 (14)	87 (13)	56 (9)	
≥2	93 (3)	5 (2)	25 (7)	21 (3)	17 (3)	
**Bodily changes**						
No	2445 (75)	188 (62)	212 (59)	506 (75)	499 (79)	<0.001
Yes	830 (25)	114 (38)	147 (41)	169 (25)	131 (21)	
**Birth-related symptoms**						
No	3238 (99)	288 (95)	337 (94)	645 (96)	607 (96)	<0.001
Yes	37 (1)	14 (5)	22 (6)	30 (4)	23 (4)	
**Breastfeeding difficulties**						
No	3147 (96)	291 (96)	334 (93)	643 (95)	605 (96)	0.08
Yes	128 (4)	11 (4)	25 (7)	32 (5)	25 (4)	
**Severe tiredness/ Fatigue**						
No	2942 (90)	273 (90)	316 (88)	615 (91)	558 (89)	0.46
Yes	333 (10)	29 (10)	43 (12)	60 (9)	72 (11)	

^a^Numbers may not sum to total because some data missing.

^b^Women who reported “the blues” were added to this group.

### Univariate analyses

In relation to mode of birth, women who had forceps-assisted vaginal births were more likely to report symptoms of depression (11%), anxiety (17%) and PTSD-type symptoms (1 symptom, 24%; 2 or 3 symptoms, 9%) at 1 month after birth than women who had unassisted vaginal births (Table 2). There were no differences between the groups for “the blues”. At 3 months after birth, women who had experienced a forceps-assisted vaginal birth were more likely to report two or more PTSD-type symptoms (7% compared with 2% or 3% for the other groups) but there were no differences between the groups on any of the other psychological outcomes (Table 3).

Physical symptoms including bodily changes were more prevalent at 1 month after birth among women who had ventouse (46%) and forceps-assisted vaginal births (43%) than women in the other groups. Birth-related symptoms (30%), breastfeeding difficulties (20%) and fatigue (32%) were also more likely at 1 month after birth amongst women who had a forceps-assisted vaginal birth than women who had unassisted vaginal births (Table 2). Although bodily changes and birth-related symptoms were higher at 3 months for those who had assisted vaginal births, there were no other differences between the groups on any of the other physical outcomes (Table 3).

### Multivariate analyses

#### Psychological symptoms by mode of birth

Unplanned caesarean section births, which may include both urgent and non-urgent procedures, were marginally associated with an increased risk of developing two or more PTSD-type symptoms (e.g. flashbacks, sleep problems unrelated to the baby) at 1 month after birth (OR = 1.52; 95% CI: 1.01-2.29) than women who had unassisted vaginal births, but there was no association at 3 months (OR = 1.16; 95% CI: 0.67-2.01). Although the associations were not significant, women who had a forceps-assisted vaginal birth had a somewhat greater risk of symptoms of anxiety at 1 month after birth (OR= 1.30; CI: 0.90-1.89) than women who had unassisted vaginal births. There was no association between a forceps-assisted vaginal birth and symptoms of anxiety at 3 months, however, the risk of PTSD-type symptoms remained significantly increased (Table 4: OR = 1.86; 95% CI: 1.06-3.24).

**Table 4:** Results from logistic regression model: Association between psychological symptoms, bodily changes, birth-related symptoms, breastfeeding, severe fatigue and mode of birth at 3 months after birth

	**Ventouse-assisted Adjusted**	**Forceps-assisted Adjusted**	**UnplannedCaesarean Adjusted**	**Planned Caesarean Adjusted**
	**OR**^**a**^**(95% CI)**	**OR**^**a**^**(95% CI)**	**OR**^**a**^**(95% CI)**	**OR**^**a**^**(95% CI)**
**Depression**				
No	1	1	1	1
Yes	0.85 (0.49-1.48)	0.99 (0.62-1.58)	0.93 (0.62-1.37)	0.89 (0.59-1.34)
**Anxiety**				
No	1	1	1	1
Yes	1.17 (0.63-2.16)	1.05 (0.62-1.79)	1.40 (0.90-2.17)	1.32 (0.83-2.09)
**PTSD symptoms**				
No	1	1	1	1
1	1.18 (0.80-1.74)	1.25 (0.87-1.79)	1.36 (1.02-1.82)	0.85 (0.61-1.17)
≥2	0.46 (0.17-1.20)	1.86 (1.06-3.24)	1.16 (0.67-2.01)	0.88 (0.48-1.63)
**Bodily changes**				
No	1	1	1	1
Yes	1.46 (1.12-1.92)	1.56 (1.21-2.01)	0.76 (0.61-0.95)	0.71 (0.56-0.89)
**Birth -related symptoms**				
No	1	1	1	1
Yes	4.31 (2.16-8.62)	4.89 (2.68-8.94)	4.21 (2.44-7.27)	4.36 (2.50-7.62)
**Breastfeeding difficulties**				
No	1	1	1	1
Yes	0.63 (0.33-1.21)	1.07 (0.65-1.77)	0.87 (0.56-1.35)	0.97 (0.61-1.53)
**Severe tiredness/ Fatigue**				
No	1	1	1	1
Yes	0.86 (0.55-1.34)	0.88 (0.59-1.31)	0.79 (0.56-1.10)	1.10 (0.81-1.51)

*Note.* The reference group is unassisted vaginal births.

^a^Adjusted for age (in years), parity (primiparous, multiparous), age on leaving full-time education (<16, 17–18, ≥19), IMD (quintiles), ethnicity (white, other) and all other birth-related outcomes presented in the table.

#### Physical symptoms by mode of birth

In comparison to women who had an unassisted vaginal birth, birth-related symptoms were increased at all three time points for the other birth groups, and were particularly high among the forceps-assisted vaginal births (Table 4: 3 month OR = 4.89; 95% CI: 2.68-8.94). At 3 months, women who had a forceps- or ventouse-assisted vaginal birth had a greater risk of bodily changes and women who had caesarean section births were less likely to report bodily changes (e.g. stress incontinence, backache, painful intercourse) at 10 days, 1 month and 3 months. No association was found between breastfeeding and mode of birth at any time point.

## Discussion

Mode of birth may have a strong association with women’s psychological and physical outcomes in the first few months after birth. As expected, women’s symptoms were highest at 10 days after birth, and the health of most women improved emotionally and physically over the following 3 months. However, some outcomes appeared to be related to the type of delivery experienced, particularly when adjusting for sociodemographic variables and the other psychological and physical symptoms present at each time point. Specifically, women who had forceps-assisted vaginal births and unplanned caesareans appeared to have poor health and psychological wellbeing after birth. The health of women who had ventouse-assisted births appeared to be somewhat compromised, while the health of women who had unassisted vaginal births and planned caesareans did not appear to be influenced by the birth process.

Women who had a forceps-assisted vaginal birth were most likely to have ongoing psychological problems after childbirth. These women were more likely to report two or more PTSD-type symptoms at 3 months, and this may be explained by the fact that their labours were more likely to have been longer, and there may have been concern about their labour or their baby as indicated by the higher levels of constant electronic monitoring reported. A lack of control, worry and an intense period of anxiety or uncertainty during labour and birth may explain the psychological symptoms reported by these women. This, in combination with other individual factors related to the mode of birth, such as a woman’s labour and birth expectations may also predispose women to stress-related symptoms after birth, as well as factors occurring after the birth, including a lack of postnatal follow-up with a health professional when this might have been helpful [27]. In our study, more than half (58%) of women who had a forceps-assisted vaginal birth reported that they spoke about their labour with a health professional. However, of those women who didn’t speak to a health professional, 43% would have liked to speak to someone. While health professionals may be more aware of the physical morbidity for women after childbirth, the ongoing psychological issues may be less clear or alternatively, perceived as more difficult to treat or beyond their area of expertise. Referral pathways need to be in place to address this point [29].

Although caesareans section births are often described as resulting in poorer postnatal psychological outcomes for women, it seems that the outcomes for women depend on whether the caesarean section is planned or not. Women having unplanned caesarean section births were marginally more likely to report PTSD-type symptoms, however, there was no association between PTSD-type symptoms and planned caesarean section births in our study. These findings are consistent with others [18-20] and are important for understanding the individual and social factors that may influence women’s postnatal psychological wellbeing. For a proportion of women it appears that there may be benefits of a planned caesarean birth, especially for those with health problems, previous complications or adverse experiences [14,30]. However, a large study of 55,000 Norwegian women did not find an association between mode of birth and a measure of emotional distress at 6 months postpartum [4], which suggests that symptoms of anxiety in new mothers may attenuate significantly with time. On the other hand, in our study depression was not associated with mode of birth, and this may suggest the need to distinguish between symptoms of anxiety and depression rather than using general measures of distress. It is possible that because our study differentiated between assisted vaginal births (forceps vs. ventouse) when assessing women’s psychological outcomes, this may have contributed to the difference in findings.

Birth related symptoms, such as painful stitches and wound infections after birth, were problematic for women during the first 3 months after birth. Those who had forceps-assisted vaginal births were more likely to report problems, and this may be explained by the high rates of episiotomy and third and fourth degree tears requiring perineal repair in this group. Consistent with previous research [7,9-11], the risk of bodily changes such as stress incontinence and backache were higher amongst women who had assisted vaginal births whereas these symptoms were less likely to affect women who had operative births.

This is one of the few studies to use population-based data to examine women’s physical and psychological outcomes after childbirth whilst taking into account co-occurring conditions and other key sociodemographic variables. As has been reported previously, women’s physical and psychological outcomes are important to take into account when examining their overall postnatal health and wellbeing [22]. In our study, we also found that women’s risk of postnatal physical symptoms was reduced when adjusting for co-occurring psychological symptoms. Our results suggest that both the psychological and physical domains need to be assessed in terms of understanding the factors influencing the duration and severity of problems affecting women’s postnatal health and wellbeing.

Although our findings are based on data from a large, national sample of women, a limitation of the study was the participation rate of 55%, a rate which is similar to that increasingly found in epidemiological studies [31]. Because women who did not participate in this study were more likely to be younger, living in a more disadvantaged area and from a BME background, and because these groups tend to have higher rates of poor health and wellbeing generally, our study may underestimate the magnitude of physical and psychological outcomes. However, the similarities between our results and those of previous studies strengthen our conclusions. Another limitation of this study was the reliance on women’s retrospective self-reports of their health and wellbeing after birth. However, the timing of survey completion and return was close enough to the birth for accuracy as far as mode of delivery and other aspects of care were concerned, and for symptom reporting at three months post-partum. Nevertheless, pre-existing experiences and those occurring after birth may be significant in increasing women’s vulnerability to distress and maintaining the distress [32] and we cannot be certain that women’s responses were not influenced by prior events or those occurring at the time of survey completion. It is also not clear how well the symptoms recorded in our study could relate to an actual diagnosis of PTSD, and thus we cannot comment on rates of clinical levels of PTSD in our sample. However, given that our findings do reflect those of previous research carried out using smaller samples and different study designs, it seems that the items used in our survey to assess PTSD-type symptoms appear to detect disturbances in new mothers.

## Conclusions

This study provides additional evidence about the importance of mode of birth in understanding women’s postnatal health and wellbeing. Our results appear to suggest that there are unique issues for women according to the mode of birth, and thus future analyses should be aware of the potential differences in wellbeing among women who have planned and unplanned caesarean births as well as ventouse and forceps-assisted vaginal births relative to unassisted vaginal births. It is concerning that women who have a forceps-assisted vaginal birth are more likely to report ongoing psychological difficulties, but are not being offered the appropriate level of healthcare support. Because these women were mostly likely to report poorer psychological and physical health following childbirth, at the same time as adjusting to life with a new baby, it may be important for health professionals to initiate conversations with women about their birth to give women the opportunity to discuss distressing symptoms. Consistent with NICE guidelines, women who describe PTSD-type symptoms at 1 month after birth should be formally tested for PTSD using a standard instrument (e.g. Traumatic Event Scale-B (TES-B); PTSD Symptom Scale-Self Report (PSS-SR) [33]. Women at risk should be monitored carefully by health professionals and appropriate referral pathways should be used in an effort to prevent chronic symptoms. Ongoing support, however, may be necessary, particularly in relation to further pregnancies. The development of preventative and supportive strategies during pregnancy, birth and afterwards would be an initial step towards improving the long-term reproductive health and wellbeing of these women.

## Competing interests

The authors declare that they have no competing interests.

## Authors’ contributions

The authors listed have contributed to this paper: IJR performed the statistical analysis and drafted the manuscript. MR was responsible for the concept and conduct of the UK 2010 National Maternity Survey and provided assistance in the interpretation of results and drafting the manuscript. IJR and MR have read and approved the final manuscript.

## Pre-publication history

The pre-publication history for this paper can be accessed here:

http://www.biomedcentral.com/1471-2393/12/138/prepub
